# *N*-(4-Meth­oxy­phen­yl)-*N*′-(5-nitro-1,3-thia­zol-2-yl)urea

**DOI:** 10.1107/S1600536810032186

**Published:** 2010-08-18

**Authors:** Alan J. Lough, Justin W. Hicks, John F. Valliant, Alan A. Wilson, Neil Vasdev

**Affiliations:** a Department of Chemistry, University of Toronto, 80 St George Street, Toronto, Ontario, Canada M5S 3H6; b PET Centre, Centre for Addiction and Mental Health and Department of Psychiatry, University of Toronto, 250 College Street, Toronto, Ontario, Canada M5T 1R8; c Departments of Chemistry and Chemical Biology, and Medical Physics and Applied Radiation Sciences, McMaster University, 1280 Main Street West, Hamilton, Ontario, Canada L8S 4K1

## Abstract

The title compound, C_11_H_10_N_4_O_4_S, is a derivative of *N*-(4-meth­oxy­benz­yl)-*N*′-(5-nitro-1,3-thia­zol-2-yl)urea (AR-A014418), a known glycogen synthase kinase 3β (GSK-3β) inhibitor. All non-H atoms in the mol­ecule are essentially coplanar, with an r.m.s. deviation of 0.045 Å and a maximum deviation of 0.115 (2) Å for the carbonyl O atom. In the crystal structure, mol­ecules are linked *via* N—H⋯O hydrogen bonds into one-dimensional chains along [101].

## Related literature

For background information on the preparation and activity of AR-A014418, see: Bhat *et al.* (2003 ▶); Inestrosa *et al.* (2006 ▶). For the radiolabelling procedure of AR-A014418 with carbon-11, see: Vasdev *et al.* (2005 ▶). For the crystal structure of AR-A014418, see: Vasdev *et al.* (2007 ▶).
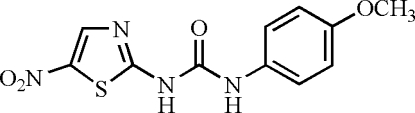


## Experimental

### 

#### Crystal data

C_11_H_10_N_4_O_4_S
*M* *_r_* = 294.29
Monoclinic, 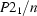

*a* = 6.8740 (3) Å
*b* = 12.5840 (7) Å
*c* = 14.6861 (5) Å
β = 101.622 (3)°
*V* = 1244.34 (10) Å^3^
*Z* = 4
Mo *K*α radiation
μ = 0.28 mm^−1^
*T* = 150 K
0.28 × 0.24 × 0.22 mm

#### Data collection

Nonius KappaCCD diffractometer
Absorption correction: multi-scan (*SORTAV*; Blessing, 1995 ▶) *T*_min_ = 0.718, *T*_max_ = 0.948
8106 measured reflections
2825 independent reflections
1979 reflections with *I* > 2σ(*I*)
*R*_int_ = 0.053

#### Refinement

*R*[*F* ^2^ > 2σ(*F* ^2^)] = 0.050
*wR*(*F* ^2^) = 0.145
*S* = 1.07
2825 reflections
190 parameters
H atoms treated by a mixture of independent and constrained refinement
Δρ_max_ = 0.38 e Å^−3^
Δρ_min_ = −0.51 e Å^−3^

### 

Data collection: *COLLECT* (Nonius, 2002 ▶); cell refinement: *DENZO-SMN* (Otwinowski & Minor, 1997 ▶); data reduction: *DENZO-SMN*; program(s) used to solve structure: *SIR92* (Altomare *et al.*, 1994 ▶); program(s) used to refine structure: *SHELXL97* (Sheldrick, 2008 ▶); molecular graphics: *PLATON* (Spek, 2009 ▶); software used to prepare material for publication: *SHELXTL* (Sheldrick, 2008 ▶).

## Supplementary Material

Crystal structure: contains datablocks I, global. DOI: 10.1107/S1600536810032186/su2205sup1.cif

Structure factors: contains datablocks I. DOI: 10.1107/S1600536810032186/su2205Isup2.hkl

Additional supplementary materials:  crystallographic information; 3D view; checkCIF report

## Figures and Tables

**Table 1:** Hydrogen-bond geometry (Å, °)

*D*—H⋯*A*	*D*—H	H⋯*A*	*D*⋯*A*	*D*—H⋯*A*
N2—H2N⋯O4^i^	0.86 (3)	1.97 (3)	2.817 (3)	168 (3)
N3—H3N⋯O3^i^	0.87 (3)	2.30 (3)	3.168 (2)	174 (2)

Symmetry code: (i) 
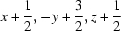
.

**Fig. 1.**
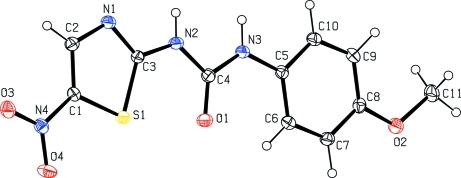
The molecular structure of the title compound with probability ellipsoids drawn at the 30% level.

**Fig. 2.**
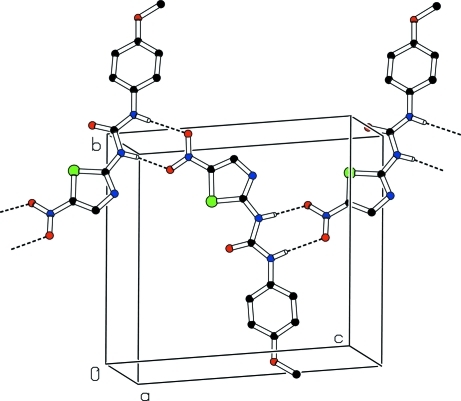
Part of the crystal structure of the title compound with hydrogen bonds drawn as dashed lines.

## supplementary crystallographic information

### Comment

*N*-(4-methoxybenzyl)-*N'-*-(5-nitro-1,3-thiazol-2-yl)urea (AR-A014418, Bhat *et al.*, 2003) is a selective glycogen synthase kinase-3β (GSK-3β) inhibitor (Inestrosa *et al.*, 2006). Our initial work on AR-A014418 was to radiolabel this compound with carbon-11 at the methoxy position for positron emission tomography (PET) imaging of brain pathologies (Vasdev *et al.*, 2005). To our surprise, [^11^C]-AR-A014418 had insignificant brain uptake in rodents despite literature precedence (Bhat *et al.*, 2003). To further understand the role of AR-A014418 and GSK-3β, a single-crystal X-ray structure of AR-A014418 was obtained (Vasdev *et al.*, 2007) and overlaid with the structural determination of the co-crystal of GSK-3β and AR-A014418 (Bhat *et al.*, 2003). For that structure, the benyzl ring was bent out of the binding pocket of the kinase. We now endeavour to explore the binding affinity of an analogous molecule which has reduced flexibility (*i.e.* the benzyl group is replaced with a phenyl group). Biological assays are underway to determine whether the increased rigidity decreases the binding affinity for GSK-3β. Data from these biological studies as well as the crystal structure determinations will assist in designing future ligands for imaging GSK-3β with PET.

The molecular structure of the title compound is shown in Fig. 1. All non-hydrogen atoms in the molecule are essentially co-planar with a r.m.s. deviation of 0.045 Å and a maximum deviation of 0.115 (2)Å for atom O1.

In the crystal symmetry related molecules are linked via N—H···O hydrogen bonds, to form one-dimensional chains propagating along [101] (Table 1, Fig. 2).

### Experimental

The title compound, C_11_H_10_N_4_O_4_S, was obtained by heating equimolar amounts of 2-amino-5-nitrothiazole (0.65 mmol) and 4-methoxyphenyl isocyanate (0.65 mmol) in dry *N*,*N*-dimethyl formamide (5 ml) in a Biotage Initiator Microwave for 1 h at 403 K under nitrogen. Upon cooling, the reaction mixture was partitioned between ethyl acetate and water and the aqueous layer was further extracted with more ethyl acetate (15 ml). The combined organic layers were washed with brine (3 × 30 ml), dried (MgSO_4_), filtered, and concentrated prior to purification by silica chromatography (Biotage Isolera Flash system, 98% dichloromethane and 2% methanol). C_11_H_10_N_4_O_4_S was obtained as a red solid in 39% yield (not optimized). X-ray quality crystals were obtained by slow evaporation of a solution of the title compound in ethyl acetate/hexane (50/50) containing 5% ethanol. ^1^H NMR (DMSO d_6_, 400 MHz) δ 11.75 (s, 1 H, NH), 9.28 (s, 1H, NH), 8.53 (s, 1H, thiazole), 7.41 (d, *J* = 8.9 Hz, 2H, Ar), 6.91 (d, *J* = 8.9 Hz, 2H, Ar), 3.73 (s, 3H, CH_3_). HRMS (ESI) m/*z* calcd for C_11_H_11_N_4_O_4_S, 295.0495; found 295.0483 (*M*^+^+H), m.p. = 454–456 K.

### Refinement

H atoms bonded to C atoms were placed in calculated positions with C—H = 0.95Å or 0.98Å for methyl groups and included in the refinement with *U*_iso_(H) = 1.2*U*_eq_(C) or 1.2*U*_eq_(C_methyl_). H atoms bonded to N atoms were refined independently with isotropic displacement parameters.

### Crystal data

**Table:**

C_11_H_10_N_4_O_4_S	*F*(000) = 608
*M**_r_* = 294.29	*D*_x_ = 1.571 Mg m^−^^3^
Monoclinic, *P*2_1_/*n*	Melting point: 454 K
Hall symbol: -P 2yn	Mo *K*α radiation, λ = 0.71073 Å
*a* = 6.8740 (3) Å	Cell parameters from 3592 reflections
*b* = 12.5840 (7) Å	θ = 2.6–27.5°
*c* = 14.6861 (5) Å	µ = 0.28 mm^−^^1^
β = 101.622 (3)°	*T* = 150 K
*V* = 1244.34 (10) Å^3^	Block, red
*Z* = 4	0.28 × 0.24 × 0.22 mm

### Data collection

**Table:**

Nonius KappaCCD diffractometer	2825 independent reflections
Radiation source: fine-focus sealed tube	1979 reflections with *I* > 2σ(*I*)
graphite	*R*_int_ = 0.053
Detector resolution: 9 pixels mm^-1^	θ_max_ = 27.5°, θ_min_ = 2.8°
φ scans and ω scans with κ offsets	*h* = −8→8
Absorption correction: multi-scan (*SORTAV*; Blessing, 1995)	*k* = −14→16
*T*_min_ = 0.718, *T*_max_ = 0.948	*l* = −17→19
8106 measured reflections	

### Refinement

**Table:**

Refinement on *F*^2^	Primary atom site location: structure-invariant direct methods
Least-squares matrix: full	Secondary atom site location: difference Fourier map
*R*[*F*^2^ > 2σ(*F*^2^)] = 0.050	Hydrogen site location: inferred from neighbouring sites
*wR*(*F*^2^) = 0.145	H atoms treated by a mixture of independent and constrained refinement
*S* = 1.07	*w* = 1/[σ^2^(*F*_o_^2^) + (0.0755*P*)^2^ + 0.3053*P*] where *P* = (*F*_o_^2^ + 2*F*_c_^2^)/3
2825 reflections	(Δ/σ)_max_ < 0.001
190 parameters	Δρ_max_ = 0.38 e Å^−^^3^
0 restraints	Δρ_min_ = −0.51 e Å^−^^3^

### Special details

**Table:**

Geometry. All e.s.d.'s (except the e.s.d. in the dihedral angle between two l.s. planes) are estimated using the full covariance matrix. The cell e.s.d.'s are taken into account individually in the estimation of e.s.d.'s in distances, angles and torsion angles; correlations between e.s.d.'s in cell parameters are only used when they are defined by crystal symmetry. An approximate (isotropic) treatment of cell e.s.d.'s is used for estimating e.s.d.'s involving l.s. planes.
Refinement. Refinement of *F*^2^ against ALL reflections. The weighted *R*-factor *wR* and goodness of fit *S* are based on *F*^2^, conventional *R*-factors *R* are based on *F*, with *F* set to zero for negative *F*^2^. The threshold expr ession of *F*^2^ > σ(*F*^2^) is used only for calculating *R*-factors(gt) *etc*. and is not relevant to the choice of reflections for refinement. *R*-factors based on *F*^2^ are statistically about twice as large as those based on *F*, and *R*- factors based on ALL data will be even larger.

### Fractional atomic coordinates and isotropic or equivalent isotropic displacement parameters (Å^2^)

**Table:**

	*x*	*y*	*z*	*U*_iso_*/*U*_eq_	
S1	0.12900 (8)	0.68571 (5)	0.41433 (3)	0.0246 (2)	
O1	0.2254 (2)	0.48461 (14)	0.47569 (10)	0.0306 (4)	
O2	0.5006 (3)	0.00530 (13)	0.62455 (11)	0.0340 (4)	
O3	−0.0126 (3)	0.97318 (14)	0.33409 (11)	0.0347 (4)	
O4	0.0063 (3)	0.82488 (15)	0.25900 (11)	0.0367 (5)	
N1	0.2026 (3)	0.79137 (16)	0.57084 (12)	0.0277 (5)	
N2	0.2668 (3)	0.61085 (16)	0.58904 (13)	0.0268 (5)	
H2N	0.327 (4)	0.627 (2)	0.6449 (19)	0.042 (8)*	
N3	0.3514 (3)	0.44014 (16)	0.62860 (13)	0.0253 (5)	
H3N	0.380 (4)	0.466 (2)	0.6845 (19)	0.037 (8)*	
N4	0.0257 (3)	0.87648 (16)	0.33258 (13)	0.0270 (5)	
C1	0.0930 (3)	0.82155 (19)	0.41566 (14)	0.0232 (5)	
C2	0.1399 (3)	0.8632 (2)	0.50308 (15)	0.0267 (5)	
H2A	0.1292	0.9369	0.5153	0.032*	
C3	0.2033 (3)	0.69504 (18)	0.53334 (15)	0.0224 (5)	
C4	0.2790 (3)	0.50759 (19)	0.55815 (15)	0.0243 (5)	
C5	0.3832 (3)	0.32879 (18)	0.62462 (15)	0.0223 (5)	
C6	0.3583 (3)	0.2717 (2)	0.54193 (15)	0.0245 (5)	
H6A	0.3153	0.3064	0.4839	0.029*	
C7	0.3970 (3)	0.1640 (2)	0.54544 (15)	0.0258 (5)	
H7A	0.3791	0.1245	0.4892	0.031*	
C8	0.4620 (3)	0.11186 (19)	0.63008 (15)	0.0253 (5)	
C9	0.4842 (4)	0.1688 (2)	0.71214 (16)	0.0281 (6)	
H9A	0.5265	0.1342	0.7702	0.034*	
C10	0.4439 (4)	0.2773 (2)	0.70889 (16)	0.0277 (5)	
H10A	0.4583	0.3164	0.7651	0.033*	
C11	0.5519 (4)	−0.0525 (2)	0.70992 (17)	0.0360 (6)	
H11A	0.5715	−0.1276	0.6965	0.054*	
H11B	0.4446	−0.0460	0.7445	0.054*	
H11C	0.6749	−0.0236	0.7472	0.054*	

### Atomic displacement parameters (Å^2^)

**Table:**

	*U*^11^	*U*^22^	*U*^33^	*U*^12^	*U*^13^	*U*^23^
S1	0.0269 (3)	0.0260 (4)	0.0198 (3)	0.0001 (2)	0.0024 (2)	−0.0017 (2)
O1	0.0405 (10)	0.0273 (10)	0.0225 (9)	0.0016 (8)	0.0028 (7)	−0.0024 (7)
O2	0.0482 (11)	0.0242 (9)	0.0304 (9)	0.0047 (8)	0.0097 (8)	0.0018 (7)
O3	0.0418 (11)	0.0279 (10)	0.0335 (10)	0.0046 (8)	0.0057 (8)	0.0080 (8)
O4	0.0450 (11)	0.0437 (12)	0.0193 (8)	0.0073 (9)	0.0015 (7)	−0.0019 (8)
N1	0.0368 (12)	0.0251 (11)	0.0214 (10)	0.0020 (9)	0.0059 (8)	−0.0009 (8)
N2	0.0352 (12)	0.0240 (11)	0.0205 (10)	0.0011 (9)	0.0040 (8)	−0.0033 (9)
N3	0.0315 (11)	0.0238 (11)	0.0204 (10)	0.0003 (9)	0.0048 (8)	−0.0014 (9)
N4	0.0239 (11)	0.0318 (12)	0.0250 (10)	0.0008 (9)	0.0041 (8)	0.0044 (9)
C1	0.0226 (12)	0.0249 (13)	0.0223 (11)	0.0005 (10)	0.0049 (9)	0.0036 (10)
C2	0.0310 (13)	0.0242 (13)	0.0246 (12)	0.0003 (10)	0.0046 (9)	−0.0005 (10)
C3	0.0241 (12)	0.0231 (13)	0.0206 (11)	−0.0022 (9)	0.0063 (8)	−0.0015 (9)
C4	0.0243 (12)	0.0251 (13)	0.0247 (12)	−0.0007 (10)	0.0079 (9)	−0.0009 (10)
C5	0.0206 (11)	0.0247 (13)	0.0226 (11)	−0.0003 (9)	0.0070 (8)	0.0002 (10)
C6	0.0233 (12)	0.0285 (14)	0.0219 (11)	0.0006 (10)	0.0051 (9)	0.0007 (10)
C7	0.0253 (12)	0.0293 (14)	0.0233 (11)	−0.0025 (10)	0.0063 (9)	−0.0030 (10)
C8	0.0257 (12)	0.0214 (12)	0.0309 (12)	0.0006 (10)	0.0105 (9)	0.0002 (10)
C9	0.0310 (13)	0.0314 (14)	0.0231 (11)	0.0006 (11)	0.0087 (9)	0.0044 (10)
C10	0.0338 (14)	0.0273 (13)	0.0235 (12)	0.0006 (11)	0.0097 (10)	−0.0020 (10)
C11	0.0477 (16)	0.0261 (14)	0.0345 (14)	0.0045 (12)	0.0089 (11)	0.0074 (11)

### Geometric parameters (Å, °)

**Table:**

S1—C3	1.723 (2)	C1—C2	1.364 (3)
S1—C1	1.728 (2)	C2—H2A	0.9500
O1—C4	1.227 (3)	C5—C10	1.385 (3)
O2—C8	1.373 (3)	C5—C6	1.391 (3)
O2—C11	1.431 (3)	C6—C7	1.381 (3)
O3—N4	1.246 (3)	C6—H6A	0.9500
O4—N4	1.245 (2)	C7—C8	1.397 (3)
N1—C3	1.332 (3)	C7—H7A	0.9500
N1—C2	1.349 (3)	C8—C9	1.384 (3)
N2—C3	1.356 (3)	C9—C10	1.391 (3)
N2—C4	1.384 (3)	C9—H9A	0.9500
N2—H2N	0.86 (3)	C10—H10A	0.9500
N3—C4	1.353 (3)	C11—H11A	0.9800
N3—C5	1.421 (3)	C11—H11B	0.9800
N3—H3N	0.87 (3)	C11—H11C	0.9800
N4—C1	1.398 (3)		
			
C3—S1—C1	86.27 (11)	C10—C5—C6	120.0 (2)
C8—O2—C11	117.49 (19)	C10—C5—N3	116.5 (2)
C3—N1—C2	109.40 (18)	C6—C5—N3	123.5 (2)
C3—N2—C4	124.68 (19)	C7—C6—C5	119.0 (2)
C3—N2—H2N	115.2 (19)	C7—C6—H6A	120.5
C4—N2—H2N	118.6 (19)	C5—C6—H6A	120.5
C4—N3—C5	128.5 (2)	C6—C7—C8	121.3 (2)
C4—N3—H3N	117.8 (18)	C6—C7—H7A	119.4
C5—N3—H3N	113.6 (18)	C8—C7—H7A	119.4
O4—N4—O3	122.61 (19)	O2—C8—C9	124.7 (2)
O4—N4—C1	117.3 (2)	O2—C8—C7	115.9 (2)
O3—N4—C1	120.09 (19)	C9—C8—C7	119.4 (2)
C2—C1—N4	127.3 (2)	C8—C9—C10	119.5 (2)
C2—C1—S1	112.58 (17)	C8—C9—H9A	120.3
N4—C1—S1	120.16 (17)	C10—C9—H9A	120.3
N1—C2—C1	114.6 (2)	C5—C10—C9	120.8 (2)
N1—C2—H2A	122.7	C5—C10—H10A	119.6
C1—C2—H2A	122.7	C9—C10—H10A	119.6
N1—C3—N2	119.29 (19)	O2—C11—H11A	109.5
N1—C3—S1	117.15 (17)	O2—C11—H11B	109.5
N2—C3—S1	123.53 (17)	H11A—C11—H11B	109.5
O1—C4—N3	126.7 (2)	O2—C11—H11C	109.5
O1—C4—N2	121.2 (2)	H11A—C11—H11C	109.5
N3—C4—N2	112.05 (19)	H11B—C11—H11C	109.5

### Hydrogen-bond geometry (Å, °)

**Table:**

*D*—H···*A*	*D*—H	H···*A*	*D*···*A*	*D*—H···*A*
N2—H2N···O4^i^	0.86 (3)	1.97 (3)	2.817 (3)	168 (3)
N3—H3N···O3^i^	0.87 (3)	2.30 (3)	3.168 (2)	174 (2)

Symmetry codes: (i) *x*+1/2, −*y*+3/2, *z*+1/2.
